# Kinetics and kinematics of diabetic foot in type 2 diabetes mellitus with and without peripheral neuropathy: a systematic review and meta-analysis

**DOI:** 10.1186/s40064-016-3405-9

**Published:** 2016-10-19

**Authors:** Animesh Hazari, Arun G. Maiya, K. N. Shivashankara, Ioannis Agouris, Ashma Monteiro, Radhika Jadhav, Sampath Kumar, C. G. Shashi Kumar, Shreemathi S. Mayya

**Affiliations:** 1 Manipal University, Manipal, India; 2 SOAHS, Manipal University, Manipal, India; 3 Department of Medicine, KMC, Manipal, India; 4 Sports and clinical Biomechanics, Robert Gordon University, Scotland, UK; 5 Department of Statistics, Manipal University, Manipal, India

## Abstract

**Background:**

Diabetes mellitus patients are at increased risk of developing diabetic foot with peripheral neuropathy, vascular and musculoskeletal complications. Therefore they are prone to develop frequent and often foot problems with a relative high risk of infection, gangrene and amputation. In addition, altered plantar pressure distribution is an important etiopathogenic risk factor for the development of foot ulcers. Thus the review on study of foot kinematic and kinetic in type 2 diabetes mellitus to understand the biomechanical changes is important.

**Methodology:**

Scientific articles were obtained using electronic databases including Science Direct, CINAHL, Springer Link, Medline, Web of Science, and Pubmed. The selection was completed after reading the full texts. Studies using experimental design with focus on biomechanics of diabetic foot were selected.

**Results:**

The meta-analysis report on gait velocity (neuropathy = 128 and non-diabetes = 131) showed that there was a significantly lower gait velocity in neuropathy participants compared to non-diabetes age matched participants at a high effect level (−0.09, 95 % CI −0.13 to 0.05; p < 0.0001). Regarding knee joint flexion range there was a significant difference between neuropathy and non-diabetes group (4.75, 95 % CI, −7.53 to 1.97, p = 0.0008).

**Conclusions:**

The systematic review with meta-analysis reported significant difference in kinematic and kinetic variables among diabetic with neuropathy, diabetic without neuropathy and non-diabetes individuals. The review also found that the sample size in some studies were not statistically significant to perform the meta-analysis and report a strong conclusion. Therefore a study with higher sample size should be done.

## Background

Diabetes is one of the most common metabolic disorders that have gained the status of a potential epidemic in India. Although the impact of the disease has been seen worldwide, more than 62 million individuals have been reported suffering with type 2 diabetes mellitus in India (Kumar et al. 2013). The prevalence of diabetes is predicted to double globally from 171 million in 2000 to 366 million in 2030 with the maximum increase in India (Wild et al. 2004). Also people with type 2 diabetes mellitus are at increased risk of peripheral arterial disease and peripheral neuropathy (Sawacha et al. 2009). The prevalence of peripheral neuropathy (DPN) among type 2 Diabetics within Indian population has been reported as 33.33 % (Pawde et al. 2013).

Diabetic peripheral neuropathy (DPN) is the most commonly seen long-term diabetes complication, involved in the pathogenesis of diabetic foot (Sawacha et al. 2009; Yavuzer et al. 2006). It affects sensory, motor and autonomic nerves that lead to progressive degeneration and loss of nerve fibers. In clinical practice, DPN is routinely assessed with changes in temperature, perception threshold, vibration and other neurological, musculoskeletal and vascular complications.

Musculoskeletal complications results from motor neuropathy that include progressive atrophy of intrinsic foot muscles leading to common foot deformities like hammer toes, claw toes, hallux valgus and prominent metatarsal heads. As a consequence, plantar pressure distribution is altered leading to higher risk of foot ulceration. High plantar pressure is an important etiopathogenic risk factor for the development of foot ulcers (Wang et al. 2015). Also diabetic foot ulceration is reported to be associated with frequent lower extremity amputation (Pham et al. 2000). However risk of ulcers can be predicted by biomechanical parameters which are determinative (Ahroni et al. 1999).

### Need for the review

From the previous studies it is evident that the prevalence of type 2 diabetes mellitus in India is high. However foot complications are the most ignored aspect. Though the basic screening of diabetic foot is practiced in many clinical settings, a complete biomechanical assessment of diabetic foot is still lacking in India. Therefore considering the higher number of individuals suffering from type 2 diabetes mellitus and its potential harm, the biomechanical assessment of foot could be highly useful to prevent future foot complications. This emphasises the need of the proposed study. The comprehensive analysis of foot biomechanics in type 2 diabetes patients could be an important clinical tool for early screening and prevention of diabetic foot complications thereby reducing amputations. Apart from these, the previous researchers showed lesser degree of agreement among themselves while reporting kinematics and kinetics of diabetic foot. Few studies reported that walking speed of neuropathic individuals in type 2 diabetes mellitus is slower when compared to non-neuropathy and non-diabetes individuals. On the others hand some authors suggested opposite results. Thus a systematic review and meta-analysis is required to propose a strong conclusion for kinematic and kinetic variation in type 2 diabetes participants with and without neuropathy compared to a healthy non-diabetes individual.

## Methods

### Literature search strategy

Scientific articles were obtained using electronic databases including Science Direct, Cinahl, Springer Link, Medline, Web of Science, and Pubmed. The search was performed in the month of December 2015. Since the three dimensional angular kinematic analysis was introduced in the early twentieth century the search was restricted from year 2000–2015 till date (Sutherland 2001).

The following keywords and MeSH headings were used:

1. Type 2 Diabetes Mellitus
2. Diabetic Peripheral Neuropathy
3. Diabetic Foot
4. Foot Biomechanics
5. Plantar pressure assessment/analysis
6. Kinetics ((and)) Kinematics Assessment
7. Gait parameters/spatiotemporal gait characteristics

Boolean Operator used—AND/OR. Full text articles in English language were selected from 2000 to 2015 to restrict the focus of the review to the most recent and advanced findings.

### Studies selection process and criteria

**Fig. 1:**
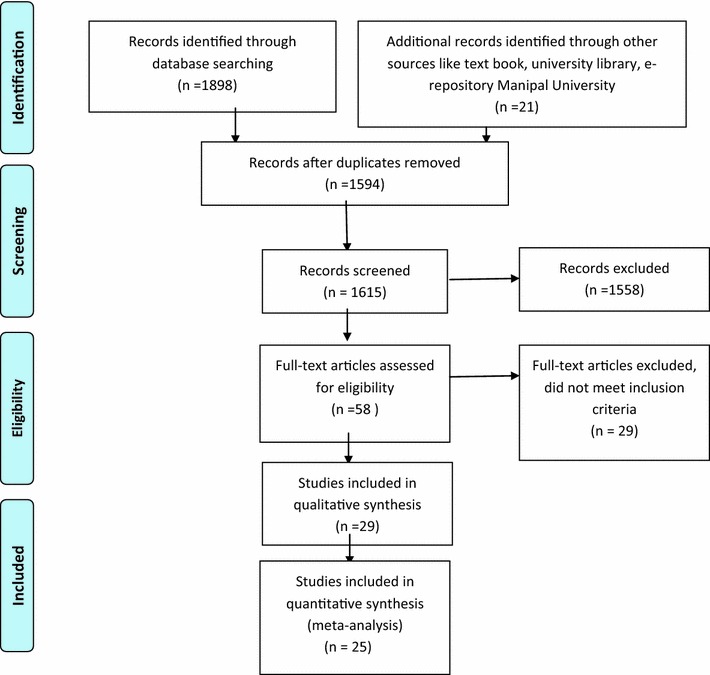
Outlines the process and step wise results from an extensive literature search

A total of 1898 records were obtained using all the search engines mentioned above that included Pubmed (n = 487), Cinahl (n = 67), Medline (n = 136), Science Direct (n = 1184), Cochrane (n = 7), Pedro (n = 3), Sports Discuss (n = 14) following which the duplicates were removed and 1594 records were obtained. This was followed by title and abstract screening under which 57 articles were pre-selected. The selection process and records have been diagrammatically shown below in Fig. 1 whereas the selected articles organized from the most recent year of publication to the most oldest based on study methods, tools to identify DPN, biomechanical tools used and outcome measures of interest have been shown in the Tables 1 and 2. The selection was completed after reading the full texts. Studies with focus on biomechanics of diabetic foot in type 2 diabetes mellitus were selected. The selection of studies was done by three authors. Following this, a consensus was obtained from all assessors in order to finally select review articles and resolve any disagreement based on the inclusion and exclusion criteria below

**Table 1:** Screening method for diabetic neuropathy

First author	Neuropathy screening	Other clinical examination
Amemiya et al. (2014)	Not specified	Not specified
Claudia et al. (2014)	1. Semmens–Weinstein 10 g monofilaments 2. Michigan neuropathy screening instrument (score ≥ 8)	Not specified
Tuna et al. (2014)	Not specified	Not specified
Raspovic (2013)	Vibration perception threshold (VPT) > 25 V in combination with a positive Neuropathy Deficit Score (NDS)	(a) Maximal isometric muscle strength of knee flexors, knee extensors and ankle dorsiflexors (b) Passive range of motion for lower limb joints
Deschamps et al. (2013)	Not specified	Not specified
Formosa et al. (2013)	Semmens–Weinstein 10 g monofilament (Neuropathy considered if one or more out of 5 sites were insensate)	Not specified
Melai et al. (2011)	Standardized neurological examination	Not specified
Gomes et al. (2011)	Michigan neuropathy screening instrument > 3/15 (questionnaire) and score of > 4/10 (examination)	General physical examination
Ko et al. (2011)	Not specified	Not specified
Rao et al. (2010)	5.07 Semmes–Weinstein Monofilament and Vibration perception threshold of 25 V or higher	Not specified
Ko et al. (2012)	Not specified	Not specified
Saura et al. (2010)	10 g Monofilament and tuning fork of 128 Hz according to the Michigan protocol	Not specified
Anjos et al. (2010)	Not specified	Not specified
Bacarin et al. (2009)	1. Michigan Neuropathy Screening Instrument questionnaire (Score > 6) 2. 10 g Monofilament (insensitive to at least 2 sites)	Not specified
Sawacha et al. (2009)	1. Michigan neuropathy screening instrument questionnaire (> 3/15 symptoms) 2. Ankle and Patellar reflex 3. Less than 3 response for 10 sites on 10 g Semmens–Weinstein monofilament test 4. Vibration pressure threshold of > 25 V 5. Pin prick using 25/7 mm needle 6. 128 MHz tuning fork	(a) Walking on heels, (b) Strength test against manual resistance for plantar flexion/extension, knee flexion/extension, adduction/abduction and forearm and finger active movements (c) General foot assessment
Savelberg et al. (2009)	Vibration perception threshold > 25 V	Ankle and knee joint muscle strength
Guldemond et al. (2008)	1. Valk Scoring system for grade of polyneuropathy (score higher than 4 was graded as peripheral polyneuropathy) 2. Pinprick sense and light touch sense (cotton wool) 3. Vibration using 128 Hz tuning fork 4. Ankle and Knee reflex	(a) Passive ankle range of motion using a plastic goniometer
Williams et al. (2007)	5.07 Semmes–Weinstein Monofilament and Vibration pressure threshold > 25 V	(a) Joint stiffness testing (b) Sensation on plantar aspect of the feet using Birke and Sims (1986)
Yavuz et al. (2008)	5.07 Semmes–Weinstein monofilament and a biothesiometer	Foot examination for ulcers
Yavuzer et al. (2006)	Not specified	None
Rahman et al. (2006)	Semmes–Weinstein monofilaments ranging from 3 to 10 g	None
Rao (2006)	5.07 Semmes–Weinstein monofilaments	Passive ankle range of motion and stiffness
Zimny et al. (2004)	Vibration pressure threshold with the calibrated Rydell-Seiffer tuning fork and the Phywe Vibratester (Threshold of 4 </8 confirmed neuropathy)	(a) Inspection of the foot (b) Palpation of the peripheral pulses
Pataky et al. (2005)	(a) Vibration Pressure Threshold (VPT) ≥ 6 measured at big toe and internal malleolus (b) Tuning Fork 128 Hz Rydel Sieffer	(a) Patellar and ankle reflex (b) Skin temperature using Thermocross
Caselli et al. (2002)	1. Stratification of participants into four groups based on the severity of neuropathy using Neuropathy Disability Score (NDS) 2. Vibration pressure threshold 3. Semmes–Weinstein monofilament	Not specified

**Table 2:** Outcome measures of interest and movement analysis tools used

Author	Movement analysis system	Outcome measures
Amemiya et al. (2014)	1. F-scan (NITTA CORPORATION, Osaka, Japan) inserted into the footwear 2. Wireless motion sensors (LOGICAL PRODUCT CORPORATION, Fukuoka, Japan)	(a) Plantar pressure (b) Gait features including amplitude of motion, gait phase balance and variability
Claudia et al. (2014)	Baropodometer (Foot Walk Pro, AM CUBE, FRANCE) at 200 Hz	(a) Gait speed, double and single stance time
Tuna et al. (2014)	Pedobarographic evaluation—A Mini-Emed pedobarography device (Novel, Munich, Germany)	(a) Peak pressure at forefoot and rear foot (b) Total plantar force (c) Forefoot and rear foot plantar force percentage (d) Total contact area and contact area percentage at forefoot and hind foot
Raspovic (2013)	1. Three-dimensional motion analysis—Vicon 512 Motion Analysis System (Oxford Metrics Ltd, Oxford, England) with six cameras operating at a sampling frequency of 100 Hz 2. A force plate (Kistler, Switzerland) embedded into a 10 m walkway operating at a sampling frequency of 400 Hz used to collect kinetic data	(a) Spatiotemporal parameters—cadence, walking speed and stride length (b) Kinematic data—stance phase range of motion: at the pelvis, hip and knee; at the ankle and first metatarsophalangeal joint in the sagittal plane; and fore- foot rotation and foot progression. Initial contact angle of the hip, knee and ankles (c) Kinetic data—maximum power and maximum moment at the hip, knee and ankle and the magnitude of the vertical ground reaction force peaks
Deschamps et al. (2013)	1. Vicon Motion System Ltd, Oxford Metrics, UK consisted of 10 T-10 cameras at 100 Hz 2. A custom made force plate (Advanced Mechanical Technology, Newton, MA, USA) covered with a pressure plate (RSscan International, Olen, Belgium)	(a) Spatio-temporal parameters of gait-stance time, swing time, walking speed and cadence (b) Peak force and % total regional impulse
Formosa et al. (2013)	Clinical examination and visual estimation	Ankle and hallux range of motion
Melai et al. (2011)	7 m wooden walkway with an imbedded pressure platform EMED-x (100 Hz, 4 sensors/cm^2^, range 0–127 N/cm^2^) or EMED-at (50 Hz, 2 sensors/cm^2^, range 0–120 N/cm^2^), Novel GmbH Inc., Munich, Germany	Plantar pressure and pressure time integral (PTI) using the Novel 10 mask division
Gomes et al. (2011)	Three biaxial electrogoniometers (Models SG110/A and SG150; Biometrics, Gwent, UK)	(a) Angular displacements of the hip, knee, and ankle joints (b) Electrical activity (Emg) of lower limb muscles
Ko et al. (2011)	1. Vicon Motion System Ltd, Oxford Metrics, UK) consisted of 10 digital cameras 2. Two staggered force platforms (Advanced Mechanical Technologies, Inc. Watertown, MA, USA at 1080 Hz	(a) Spatiotemporal parameters-walking speed, stride length, stride width (b) Range of motion for hip, knee and ankle (c) Generative and absorptive power at Hip, Knee and ankle
Rao et al. (2010)	1. Active marker system (Optotrak, NDI, Waterloo, Canada) at 120 Hz 2. Forceplate embedded in the walkway (Kistler Inc, Amherst, NY) at 360 Hz 3. Pedobarograph (EMed, Novel Inc., St Paul, MN) at 50 Hz	(a) Kinematic data—Peak motion as well as excursion for the 1st metatarsal, lateral forefoot and calcaneus (b) Kinetic data—Ankle joint plantarflexor moment and power and Plantar pressure- heel, midfoot, forefoot
Ko et al. (2012)	1. Gait-RITE™ mat (Gait-RITE CIRSystem, Inc., Havertown, PA, USA) 2. Teskan High Resolution Floor Mat System (Teskscan Inc, South Boston, MA, USA)	(a) Kinematics-walking speed (b) Gait variables on dominant limb-cadence, step length, step time, and toe out angle. Centre of force medial–lateral (MLE) and anterior-posterior excursion (APE) (c) Kinetics—Peak plantar pressure (PPP)
Saura et al. (2010)	1. Vicon^®^ system, using 4 cameras (Mcam2 at 250 Hz) 2. Force platform (AMTI^®^ OR6/6 at 1000 Hz)	(a) Vertical ground reaction force (GRF) (b) Ankle Range of Motion (ROM)
Anjos et al. (2010)	Pressure foot plate from the Footwork Analysis System, with 2704 sensors measuring 7.62 × 7.62 mm	Mean peak plantar pressure
Bacarin et al. (2009)	Pedar-X system (20 steps and a sampling rate of 50 Hz)	(a) Peak pressure (b) Pressure time integral at hallux, medial forefoot, lateral forefoot, mid foot and rear-foot
Sawacha et al. (2009)	BTS motion capture system (Six cameras, 60–120 Hz) Synchronized with two Bertec Force plates (FP4060-10) and integrated with two Imago plantar pressure system (0.64 cm^2^ resolution, 150 Hz)	1. Spatio-temporal parameters 2. Ground reaction force, centre of pressure and peak pressure
Savelberg et al. (2009)	12 m walkway Kistler type 9281A pressure platform (Novel GmbH, Munich, Germany)	(a) Gait variable-velocity (b) Ground reaction force (c) Ankle, knee and hip joint moments
Guldemond et al. (2008)	An EMED SF-4^®^ pressure sensitive platform (Novel, Munich) for barefoot plantar pressures analysis	Peak Pressure at forefoot, hallux and all five MTP joints
Williams et al. (2007)	1. 5 camera motion analysis system using Retroreflective markers 2. Force plate at 960 Hz and 10 foot strikes were taken.	(a) Joint angles at ankle and knee (b) Joint moments (c) Joint stiffness using the method described by Stefanyshyn and Nigg (1998)
Yavuz et al. (2008)	A custom-built shear and pressure platform, 80 sensors (12.5 mm_12.5mm) arranged in an 8_10 array	(a) Pressure time integral (PTI) (b) Stress time integral (STI) (c) Peak pressure (PP) (d) Anterior-posterior (AP) and Medial–lateral shear (ML) stress (e) Peak to peak AP and peak to peak ML pressure
Yavuzer et al. (2006)	1. Vicon 370 system (Vicon Oxford Metrix Limited, 14Minns Estate, West way, Oxford, OX2 OJB) 2. Two Bertec forceplates (Bertec Corp. Columbus, OH)	(a) Gait parameters-cadence, walking velocity, stride and step time, stride and step length and double support time (b) Kinematics-joint rotation angle of pelvis, hip, knee and ankle (c) Kinetics-vertical forces, momentum and power of hip, knee and ankle
Rao (2006)	1. Recording at 60 Hz using an active marker system (Optrotrak, NDI, Waterloo, Canada) 2. Force plate at 240 Hz (Kistler Inc., NY) 3. Pressure sensitive insoles (Pedar, Novel Inc., Minneapolis, MN) at 50 Hz	(a) Passive range of motion for ankle (b) Ankle joint stiffness (c) Peak pressure, peak joint moment and peak power for ankle (c) Gait parameters-walking speed, stride length (d) Joint peak power
Rahman et al. (2006)	F-Scan in-shoe pressure measurement system (Ngee Ann Polytechnic, Singapore)	(a) Peak pressure (b) Contact area (c) Percentage medial impulse
Zimny et al. (2004)	Fast Scan system (Megascan, Hannover, Germany)	(a) Range of motion for ankle and 1st metatarso-phalangeal (MTP) joint (b) Plantar pressure Integral
Pataky et al. (2005)	Force sensing resistors sensors 174^®^, International Electronics and Engineer-ing, Luxemburg	Peak plantar pressure on big toe, 1st, 3rd, 5th meta-tarsal and heel
Caselli et al. (2002)	F-Scan mat system, software version 3.711 (Teskan, Boston, MA)	(a) Passive range of motion for 1st MTP and Subtalar joint using a goniometer (b) Maximum peak pressure under forefoot and rearfoot

**Table:** .

Inclusion criteria	Exclusion criteria
Studies comparing DPN with and without neuropathy with normal individuals Barefoot biomechanical analysis Outcome measures of interest—(a) spatiotemporal parameters (walking speed, step length, stride length, etc.) (b) Kinematic variables of knee and ankle joint during stance and dynamic gait cycle: joint angle, velocity, momentum, acceleration, power etc. (c) Kinetic variables of knee and ankle joint during stance and dynamic gait cycle: GRF, Pressure, COM etc. (d) Plantar pressure using static or dynamic foot scanner, force plate	Studies that did not report at least one outcome variable of interest Studies without barefoot analysis or using any assistive devices Studies that reported subjects with previous foot ulcers Studies with neuropathy other that diabetic origin Studies without a proper and comprehensive methodology Studies that used various methods and tools for calculating the kinematic and kinetic variables other than motion analysis software or force platforms or pedography

### Search results

Figure 1 outlines the process and step wise results from an extensive literature search.

### Study quality assessment

The included studies were independently assessed by three reviewers using the quality assessment tool given by Downs and Black (1998). The overall scoring was done on 27 domains out of which 10 questions were not commonly applicable to the reviewed studies. Therefore the score was based upon 17 domains and the study was classified as poor (<7/17), fair (8–11/17) and good (>11/17) accordingly, as a simplified Downs and Black quality assessment tool (Fernando et al. 2013). For the purpose of agreement, the average score of the three assessors for each domain and overall total score has been shown in the Table 3.

**Table 3:** Study quality assessment using Downs and Black (1998)

Down and black questions	Amemiya et al. (2014)	Raspovic (2013)	Anjos et al. (2010)	Bacarin et al. (2009)	Caselli et al. (2002)	Deschamps et al. (2013)	Claudia et al. (2014)	Formosa et al. (2013)
Total score	16	10	11	12	14	11	12	03
1	Y	Y	Y	Y	Y	Y	Y	Y
2	Y	Y	Y	Y	Y	Y	Y	N
3	Y	Y	Y	Y	Y	Y	Y	N
4	NR	NR	NR	NR	NR	NR	NR	NR
5	Y	Y	Y	P	Y	Y	Y	N
6	Y	Y	Y	Y	Y	N	Y	N
7	Y	Y	N	y	Y	Y	Y	N
8	NR	NR	NR	NR	NR	NR	NR	NR
9	NR	NR	NR	NR	NR	NR	NR	NR
10	Y	Y	Y	N	Y	Y	Y	N
11	Y	UTD	UTD	Y	Y	Y	UTD	UTD
12	Y	UTD	UTD	UTD	UTD	UTD	UTD	UTD
13	N	Y	Y	Y	Y	Y	Y	UTD
14	NR	NR	NR	NR	NR	NR	NR	NR
15	NR	NR	NR	NR	NR	NR	NR	NR
16	UTD	UTD	N	UTD	UTD	N	UTD	N
17	NR	NR	NR	NR	NR	NR	NR	NR
18	Y	Y	Y	Y	Y	Y	Y	UTD
19	NR	NR	NR	NR	NR	NR	NR	NR
20	Y	Y	Y	Y	Y	UTD	Y	UTD
21	Y	UTD	Y	Y	Y	Y	Y	Y
22	Y	UTD	Y	ND	Y	Y	Y	Y
23	NR	NR	NR	NR	NR	NR	NR	NR
24	NR	NR	NR	NR	NR	NR	NR	NR
25	UTD	UTD	UTD	Y	Y	UTD	UTD	UTD
26	NR	NR	NR	NR	NR	NR	NR	NR
27	N	N	N	N	N	N	N	N

Down and black questions	Gomes et al. (2011)	Guldemond et al. (2008)	Melai et al. (2011)	Pataky et al. (2005)	Rehman	Saura et al. (2010)	Sacco et al. (2009)	Saura et al. (2010)	Sacco et al. (2009)	Seung
Total score	9	12	10	12	08	10	12	10	12	14
1	Y	Y	Y	Y	N	Y	Y	Y	Y	Y
2	Y	Y	Y	Y	Y	Y	Y	Y	Y	Y
3	Y	Y	Y	Y	Y	Y	Y	Y	Y	Y
4	NR	NR	NR	NR	NR	NR	NR	NR	NR	NR
5	Y	Y	N	Y	Y	Y	Y	Y	Y	Y
6	Y	Y	Y	Y	N	Y	Y	Y	Y	Y
7	Y	Y	Y	Y	Y	Y	Y	Y	Y	Y
8	NR	NR	NR	NR	NR	NR	NR	NR	NR	NR
9	NR	NR	NR	NR	NR	NR	NR	NR	NR	NR
10	Y	N	N	Y	N	N	N	N	N	Y
11	UTD	Y	Y	UTD	UTD	Y	Y	Y	Y	UTD
12	UTD	UTD	UTD	UTD	UTD	UTD	UTD	UTD	UTD	UTD
13	UTD	UTD	Y	Y	N	Y	Y	Y	Y	Y
14	NR	NR	NR	NR	NR	NR	NR	NR	NR	NR
15	NR	NR	NR	NR	NR	NR	NR	NR	NR	NR
16	UTD	Y	Y	UTD	N	UTD	Y	UTD	Y	Y
17	NR	NR	NR	NR	NR	NR	NR	NR	NR	NR
18	Y	Y	UTD	Y	Y	Y	Y	Y	Y	Y
19	NR	NR	NR	NR	NR	NR	NR	NR	NR	NR
20	Y	Y	Y	Y	Y	Y	N	Y	N	Y
21	UTD	Y	Y	Y	Y	UTD	Y	UTD	Y	Y
22	UTD	UTD	UTD	Y	Y	UTD	UTD	UTD	UTD	Y
23	NR	NR	NR	NR	NR	NR	NR	NR	NR	NR
24	NR	NR	NR	NR	NR	NR	NR	NR	NR	NR
25	N	Y	N	UTD	UTD	N	N	N	N	Y
26	NR	NR	NR	NR	NR	NR	NR	NR	NR	NR
27	N	N	N	N		N	N	N	N	N

Down and black questions	Savelberg et al. (2009)	Sawacha et al. (2009)	Sawacha et al. (2009)	Sawacha et al. (2009)	Sawacha et al. (2012)	Uccioli et al. (2001)	Yavuzer et al. (2006)
Total score	13	13	13	10	12	10	13
1	Y	Y	Y	N	Y	Y	Y
2	Y	Y	Y	Y	Y	Y	Y
3	Y	Y	Y	Y	Y	Y	Y
4	NR	NR	NR	NR	NR	NR	NR
5	Y	Y	Y	Y	Y	Y	Y
6	Y	Y	Y	Y	Y	Y	Y
7	Y	Y	Y	Y	Y	Y	Y
8	NR	NR	NR	NR	NR	NR	NR
9	NR	NR	NR	NR	NR	NR	NR
10	Y	N	N	N	Y	N	Y
11	UTD	Y	Y	Y	Y	UTD	UTD
12	Y	UTD	UTD	UTD	UTD	UTD	UTD
13	Y	Y	Y	Y	Y	UTD	Y
14	NR	NR	NR	NR	NR	NR	Y
15	NR	NR	NR	NR	NR	NR	Y
16	Y	N	N	Y	UTD	Y	Y
17	NR	NR	NR	NR	NR	NR	NR
18	Y	Y	Y	Y	Y	Y	Y
19	NR	NR	NR	NR	NR	NR	NR
20	Y	Y	Y	Y	Y	Y	Y
21	Y	Y	Y	UTD	Y	Y	Y
22	UTD	UTD	UTD	UTD	UTD	UTD	UTD
23	NR	NR	NR	NR	NR	NR	NR
24	NR	NR	NR	NR	NR	NR	NR
25	N	Y	Y	N	N	UTD	UTD
26	NR	NR	NR	NR	NR	NR	NR
27	N	N	N		N	N	N

Y = 1; N = 0; NR, not relevant (the study design doesn’t include these components); UTD, unable to determine

### Data extraction

The process of data extraction was accomplished by the first author with the help of a qualified statistician from the University Biostatistics department. All the studies that reported the outcome measures of interest were included for statistical analysis. However qualitative studies were only included for the critical reviews and excluded from statistical analysis.

### Statistical analysis

The descriptive statistics (SPSS v.16) was performed for the participant characteristics like age, height, weight, BMI, duration of diabetes etc. For the purpose of easy comparison and statistical analysis, the outcome measures of interest were transformed into standard units. Following this, meta-analysis using forest plot was carried out for all outcome measures that have been reported in detail in the result section below. Since the sample size in the review studies were not equally distributed and the comparison included the healthy participants, random effect model forest plot was constructed in order to compute a combined effect that estimated the mean effect of the distribution. The weight assigned under random effect model is more balanced where larger sample size studies are less likely to dominate the analysis and small studies are less likely to be trivialized (Borenstein et al. 2007). The effect size was computed using Cohen’s d. Cohen’s d score of zero was considered as no effect, whereas a result of 0–0.2 was interpreted as small effect difference, 0.2–0.8 as medium effect size and ≥0.8 a large effect difference (Fernando et al. 2013). Heterogeneity was calculated using the I^2^ statistic. Finally the results were reported as standardized mean differences with 95 % confidence intervals and p values.

## Review findings and results

### Search details

A total of 25 articles were finally selected for the review. There were various scientific reasons and grounds for excluding these records, such as inappropriate title and methods, inappropriate design, outcome measures and tools used were not appropriate, lack of diabetes classification, inappropriate data, and language other than English etc.

### Study quality

Majority of the study included in review were of good and fair quality based on the Downs and Black scoring (Table 3). However, majority of them failed to score on the 27th question. Only two studies reported about sample size calculation. Apart from these, there was a lot of variability in reporting various confounding variables (duration of disease, BMI, muscular weakness, neuropathic pain, severity of diabetic neuropathy, any musculoskeletal related joint pain, chronic ankle instability, foot and ankle deformities) pertaining to biomechanical outcomes.

### Participant characteristics

The participants in the studies were categorized into three group viz. Type 2 diabetes mellitus with neuropathy, Type 2 DM without neuropathy and non-diabetes (Control) age matched participants. The descriptive characteristics of participants have been given in Table 4. The selection criteria for neuropathy has been reported in Table 1.

**Table 4:** Demographic data of participants from included studies

Demographics	Neuropathy	Non-neuropathy	Normal
	Mean ± SD (n)	Mean ± SD (n)	Mean ± SD (n)
Age (years)	60.53 ± 8.21 (431)	52.83 ± 8.80 (385)	61.21 ± 7.3 (467)
Height (m)	1.68 ± 0.09 (108)	1.65 ± 0.08 (162)	1.66 ± 0.07 (338)
Weight (kg)	83.91 ± 15.88 (145)	77.03 ± 9.48 (125)	69.92 ± 8.98 (330)
BMI	27.36 ± 4.33 (277)	27.58 ± 4.82 (215)	24.85 ± 3.04 (156)
Disease duration	14.51 ± 8.43 (297)	12.99 ± 8.1 (181)	Not applicable

#### Participant recruitment strategy

A variety of participant recruitment sources were found among the various researchers. These included community outpatient settings, hospital settings, and volunteers. For comparison healthy control was included in some studies on a voluntary basis.

#### Screening process

Screening the participants is an important process for the diagnosis of DPN. Majority of the studies utilized Michigan Neuropathy Screening Instrument (MNSI) to determine the presence of sensory neuropathy. However Monofilament, Biothesiometer or VPT, clinical assessment was also used by few studies (Table 1). On the contrary; one study also used the nerve conduction test (NCV) to diagnose DPN (Yavuzer et al. 2006).

#### Outcome measures

Regarding the outcome measures, the variables of interest found in majority of the studies were spatiotemporal parameters, kinetics and kinematics of stance and dynamic phase. Each variable has been discussed in detail below.

#### Spatiotemporal gait parameters

##### Gait velocity

**Fig. 2:**
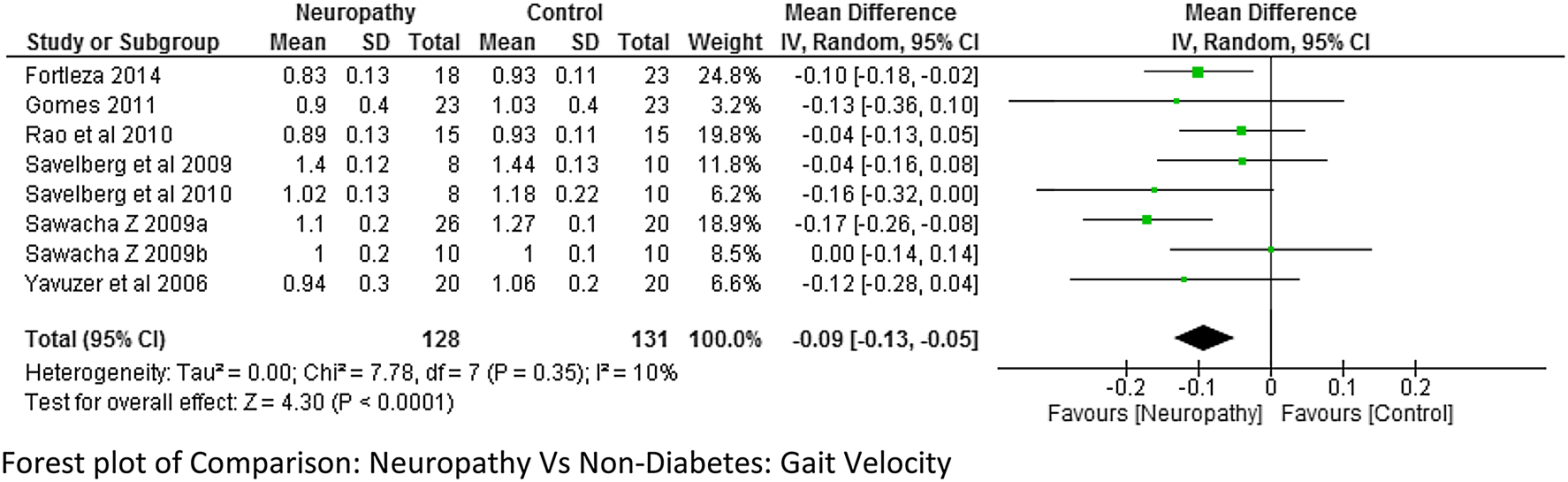
Meta-analysis showing gait velocity in non-diabetes (control) compared to diabetes with neuropathy (*negative* mean difference represents higher mean values in the second group i.e. non-diabetes)

**Fig. 3:**
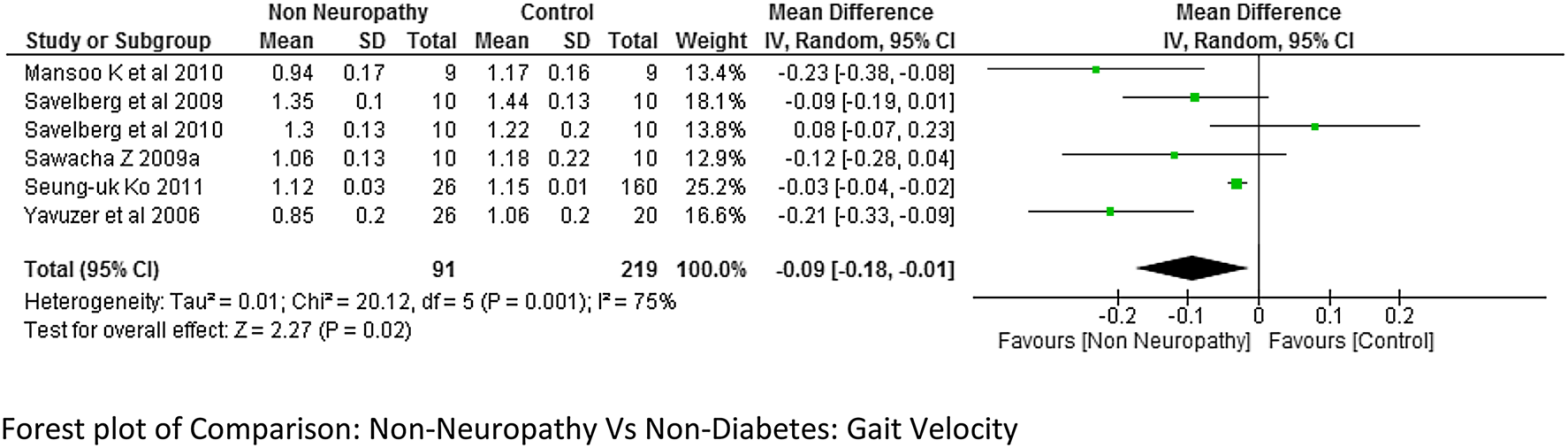
Meta-analysis showing gait velocity in non-diabetes compared to diabetes without neuropathy (*negative* mean difference represents higher mean values in the second group i.e. non-diabetes)

Walking speed/gait velocity was reported by 10 studies (Sawacha et al. 2009; Claudia et al. 2014; Gomes et al. 2011; Rao et al. 2010; Savelberg et al. 2009; Ko et al. 2011, 2012; Raspovic 2013). Out of them seven studies compared neuropathic participants with non-diabetes (normal/control) participants and the rest reported gait velocity difference between non-neuropathic and non-diabetes participants. There were four studies that reported data between both neuropathy and non-diabetic, non-neuropathy and non-diabetic (Sawacha et al. 2009; Yavuzer et al. 2006; Savelberg et al. 2009, 2010). The Meta-analysis report on gait velocity (neuropathy = 128 and non-diabetes = 131) showed that there was a significantly lower gait velocity in neuropathy participants compared to non-diabetes age matched participants at a high effect level (−0.09, 95 % CI −0.13 to 0.05; p < 0.0001). In the present study, negative combined effect suggests that the mean was higher in the second comparable group i.e. non-diabetes whereas the positive value would suggest greater mean values in the first group. The heterogeneity among the studies was less I^2^ = 10 % (Fig. 2). Also the meta-analysis report on gait velocity between non-neuropathy participants and non-diabetes participants showed greater velocity for non-diabetes group compared to the non-neuropathy group at a moderate effect level p = 0.02, however there was a high heterogeneity between the studies I^2^ = 75 % (Fig. 3).

##### Stride length and stance period

**Fig. 4:**
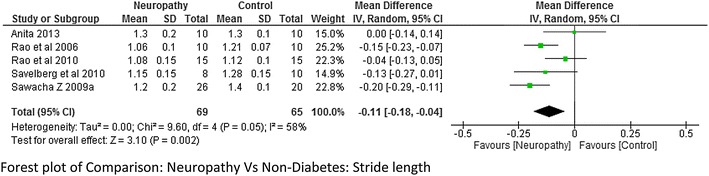
Meta-analysis showing stride length in non-diabetes compared to diabetes with neuropathy (*negative* mean difference represents higher mean values in the second group i.e. non-diabetes)

**Fig. 5:**
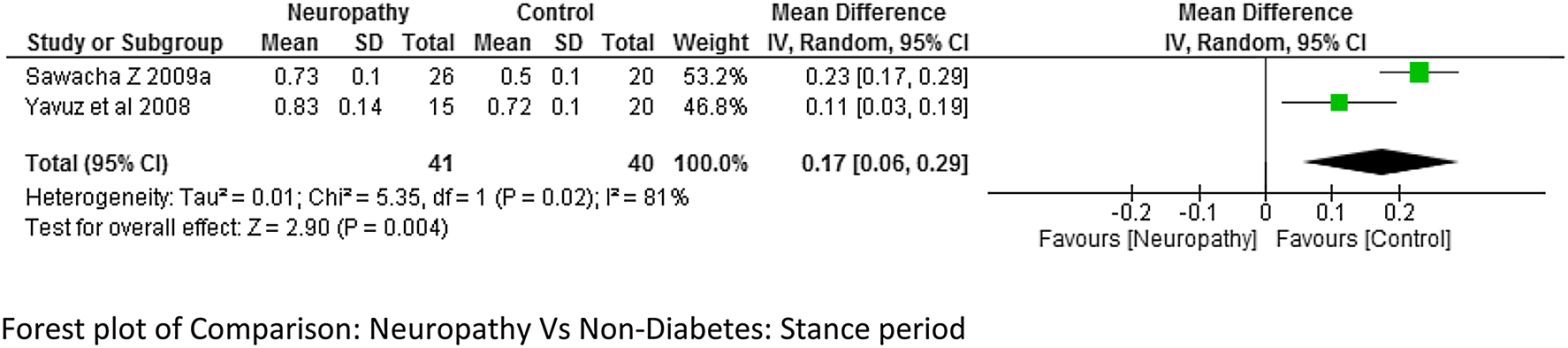
Meta-analysis showing stance period in diabetes with neuropathy compared to non-diabetes (*positive* mean difference represents higher mean values in first group i.e. diabetics with neuropathy)

**Fig. 6:**
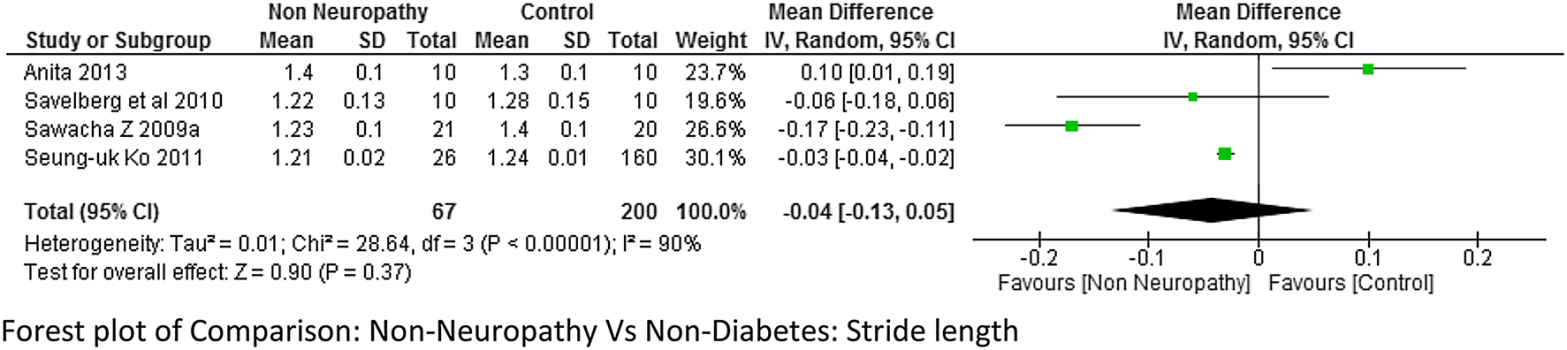
Meta-analysis showing stride length in non-diabetes compared to diabetes without neuropathy (*negative* mean difference represents higher mean values in the second group i.e. non-diabetes)

**Fig. 7:**
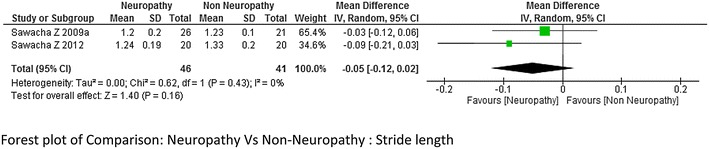
Meta-analysis showing stride length in diabetes without neuropathy compared to diabetes with neuropathy (*negative* mean difference represents higher mean values in the second group i.e. Diabetes without neuropathy)

The meta-analysis report on stride length and stance period (neuropathy = 69 and non-diabetes = 65 and neuropathy = 45 and non-diabetes = 45 respectively) from combing the data of the studies done by Sawacha et al. (2009), Rao et al. (2010), Savelberg et al. (2009), Raspovic (2013), Yavuz et al. (2008) suggested that stride length was significantly lower in neuropathic group compared to non-diabetes group, whereas stance period was significantly higher in neuropathic group. The heterogeneity among the studies for both stride length and stance period was high I^2^ = 58 and I^2^ = 81 % respectively (Figs. 4 and 5 respectively). Only two studies (Sawacha et al. 2009, 2012) reported on stride length and stance period between non-neuropathy and non-diabetes group, non-neuropathy and neuropathy group but results were not significant to support either group (Figs. 6 and 7).

##### Kinematics

**Fig. 8:**
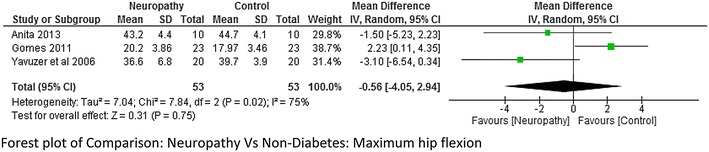
Meta-analysis report for hip flexion range between neuropathy and non-diabetes participants (results do not favor either group)

**Fig. 9:**
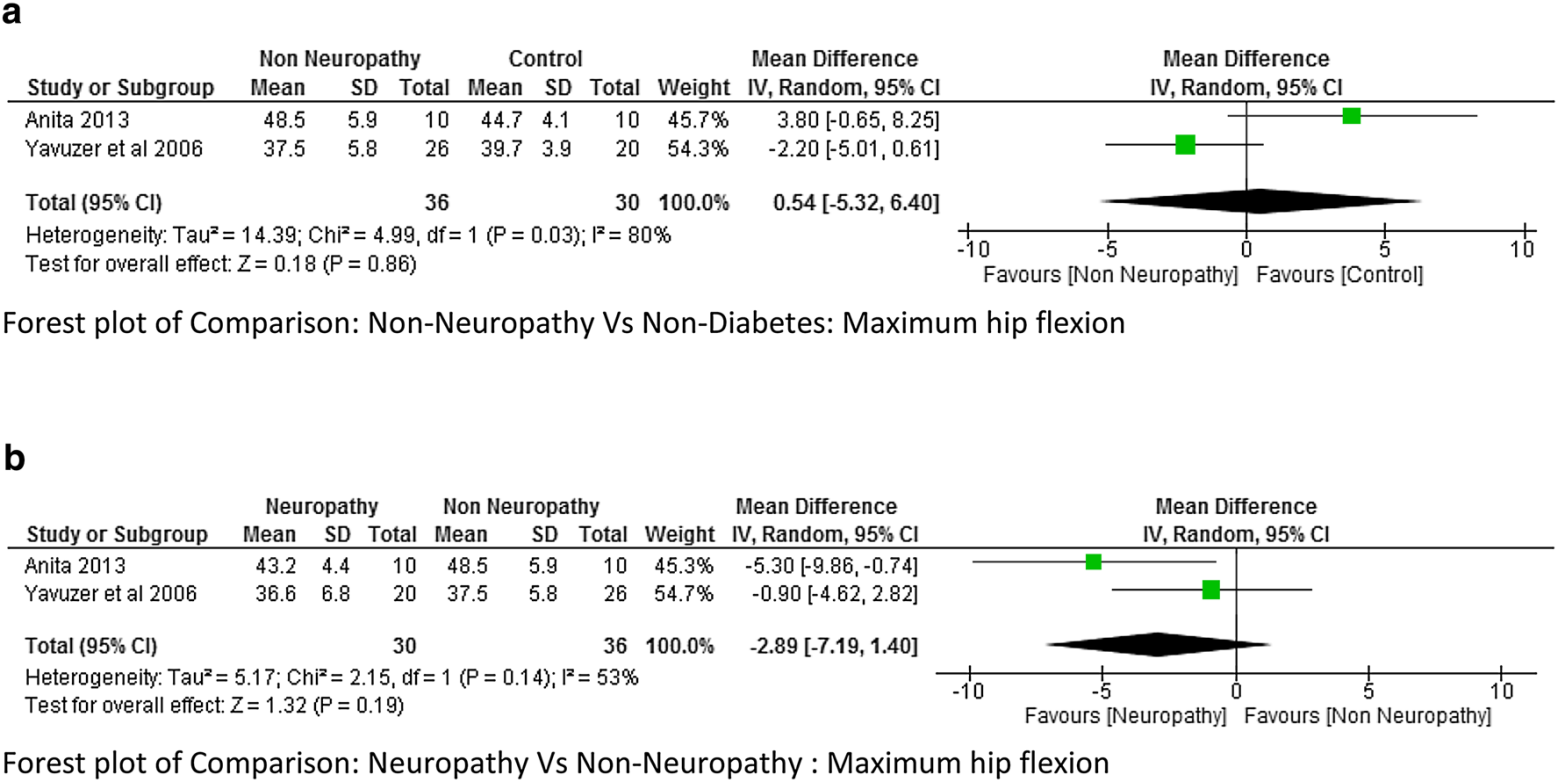
**a** Meta-analysis report for hip flexion range between non-neuropathy and non-diabetes participants (results do not favor either group). **b** Meta-analysis report for hip flexion range between non-neuropathy and neuropathy (*negative* mean difference represents higher mean values in the second group i.e. diabetes without neuropathy)

**Fig. 10:**
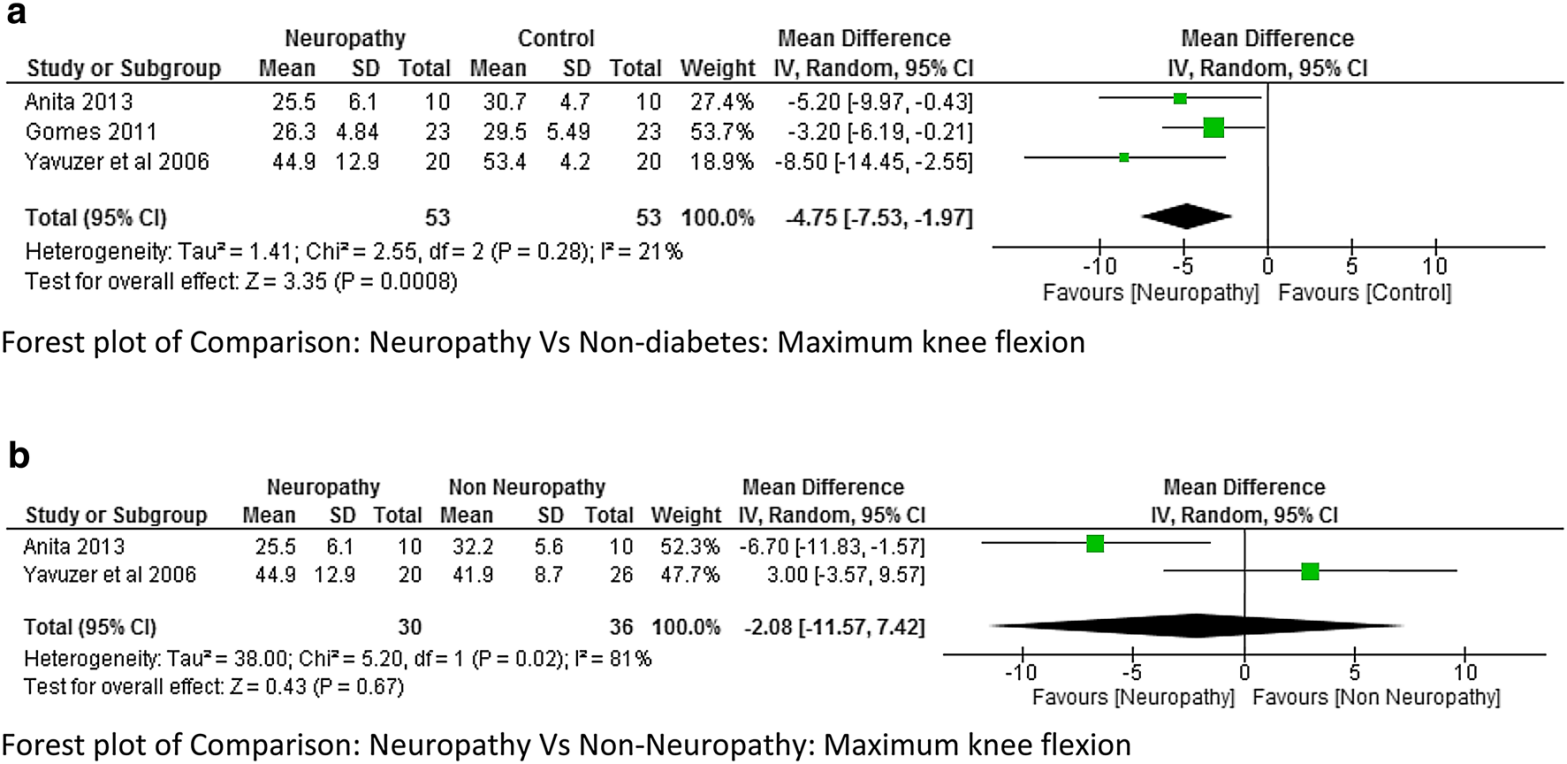
**a** Meta-analysis report for knee flexion range between neuropathy and non-diabetic participants (*negative* mean difference represents higher mean values in the second group i.e. non-diabetes). **b** Meta-analysis report for knee flexion range between neuropathy and non-neuropathy (results do not favor either group)

**Fig. 11:**
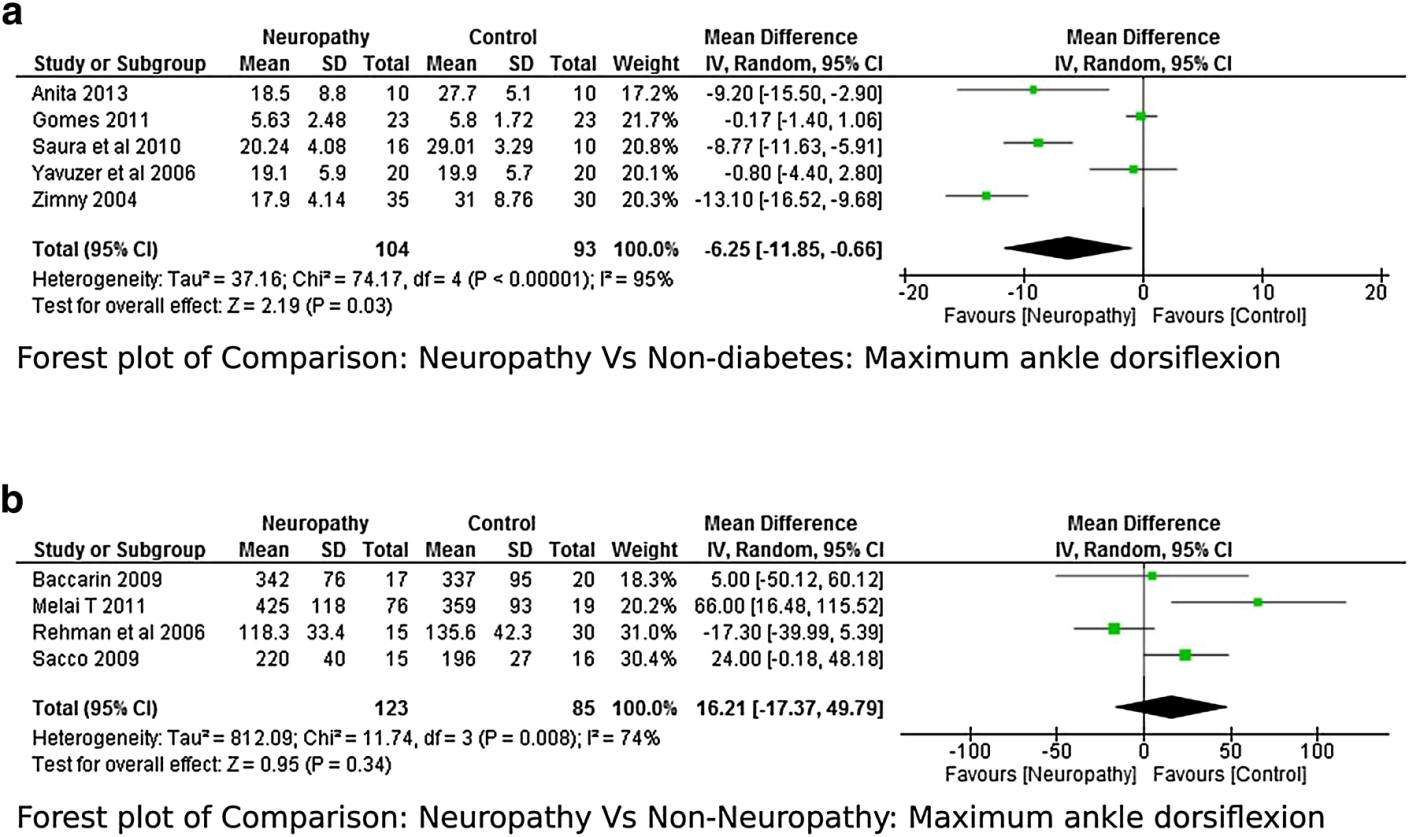
**a** Meta-analysis report for ankle dorsiflexion range between neuropathy and non-diabetic participants (*negative* mean difference represents higher mean values in the second group i.e. non-diabetes). **b** Meta-analysis report for ankle dorsiflexion range between neuropathy and non-neuropathy (results do not favor either group)

Five studies (Yavuzer et al. 2006; Gomes et al. 2011; Raspovic 2013; Saura et al. 2010; Zimny et al. 2004) reported kinematic variables like hip, knee and ankle joint range of motion. There was a lot of variability while reporting maximum hip flexion range with a higher heterogeneity I^2^ = 75 %. Two studies (Gomes et al. 2011; Raspovic 2013) found that the hip flexion range was higher in neuropathy compared to non-diabetes group whereas one study (Yavuzer et al. 2006) found it to be less, therefore meta-analysis report was not significant (Fig. 8). However no significant difference was found between Non-neuropathy and non-diabetes group, neuropathy and non-neuropathy group (Fig. 9a, b). Regarding knee joint flexion range there was a significant difference between neuropathy and non-diabetes group (Fig. 10a). The Meta-analysis report showed that maximum knee flexion angle was significantly higher in non-diabetes group at high effect level (−4.75, 95 % CI −7.53 to 1.97, p = 0.0008 and lower heterogeneity I^2^ = 21 %). However no conclusion could be drawn between neuropathy and non-neuropathy group regarding maximum knee flexion range of motion (Fig. 10b). Similarly the maximum ankle dorsiflexion angle was found to be significantly higher in non-diabetes group compared to both neuropathy and non-neuropathy group at moderate effect level, however there was a higher heterogeneity of I^2^ = 95 % (neuropathy and non-diabetes) as one study (Gomes et al. 2011) had lower mean values compared to other studies (Fig. 11a). Also similar to knee joint, no significant difference was seen at ankle dorsiflexion for neuropathy and non-neuropathy group (Fig. 11b).

## Kinetics

The kinetic variables of interest reported from the included study were plantar pressure, GRF (ground reaction force) and joint moment.

### Plantar pressure

**Fig. 12:**
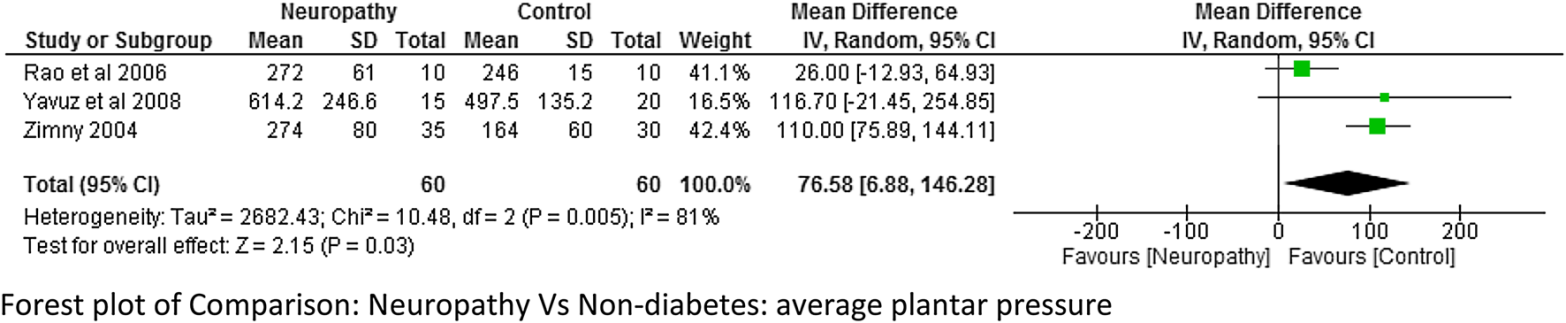
Meta-analysis report for plantar pressure between neuropathy and non-neuropathy (*positive* mean difference represents higher values in first group i.e. diabetes with neuropathy)

**Fig. 13:**
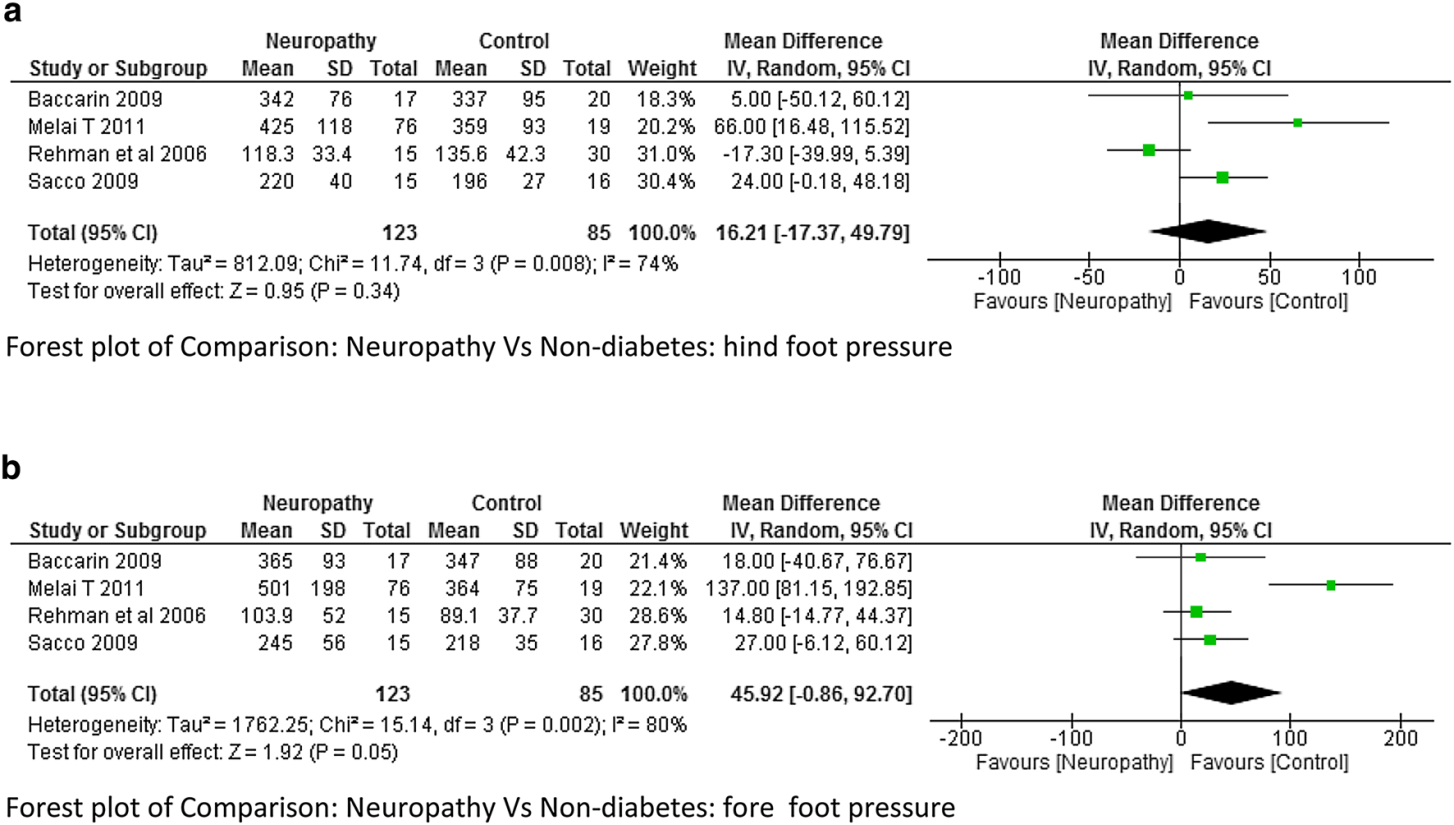
**a** Meta-analysis report for hind foot pressure between neuropathy and non-diabetic participants (results do not favor either group). **b** Meta-analysis report for fore foot pressure between neuropathy and non-diabetic participants (*positive* mean difference represents higher values in first group i.e. diabetes with neuropathy)

**Fig. 14:**
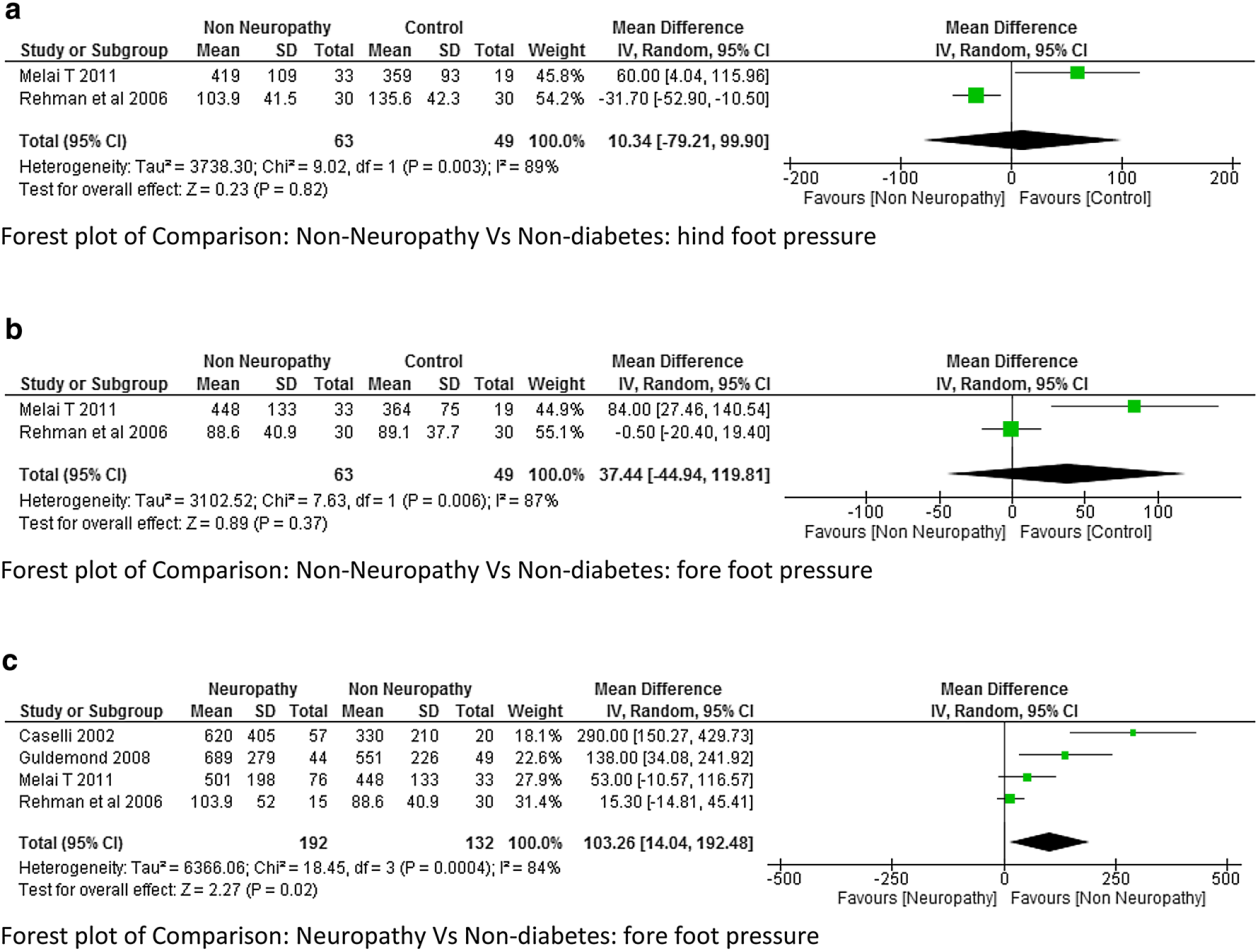
**a** Meta-analysis report for hind foot pressure between non-neuropathy and non-diabetic participants (results do not favor either group). **b** Meta-analysis report for fore foot pressure between non-neuropathy and non-diabetic participant (results do not favor either group). **c** Meta-analysis report for fore foot pressure between neuropathy and non-neuropathy (*positive* mean difference represents higher values in first group i.e. diabetes with neuropathy)

This was the most common variable studied by many authors. The plantar pressure was divided into three areas like forefoot, mid-foot and hind foot. Average plantar pressure was reported by three studies (Rao et al. 2010; Zimny et al. 2004; Yavuz et al. 2008). The meta-analysis report suggested that there was very high heterogeneity I^2^ = 81 % between neuropathy and non-diabetes group although a significant higher value of plantar pressure was seen in neuropathic group at moderate effect (p = 0.03; Fig. 12). Hind foot and mid foot pressure was reported by Bacarin et al. (2009), Melai et al. (2011), Rahman et al. (2006), Sacco et al. (2009). There was a lot of variability among the researchers while reporting mean plantar pressure in these two areas. As a result very high heterogeneity was obtained in the meta-analysis report (Fig. 13a, b). Only two studies reported the data on hind foot and fore foot pressure between non-neuropathy and non-diabetes group. The meta-analysis report was not significant with very high heterogeneity (Fig. 14a, b). It was difficult to determine which group has higher plantar pressure based on two studies (Melai et al. 2011; Rahman et al. 2006). Whereas three studies (Melai et al. 2011; Rahman et al. 2006; Caselli et al. 2002) reported hind foot and fore foot pressure between neuropathy and non neuropathy group. The meta-analysis report suggested there was no significant difference at hind foot however a significant higher value of forefoot pressure with moderate effect size (p = 0.02) was found in neuropathy group though the heterogeneity was again high I^2^ = 84 % (Fig. 14c).

### Ground reaction force (GRF)

**Fig. 15:**
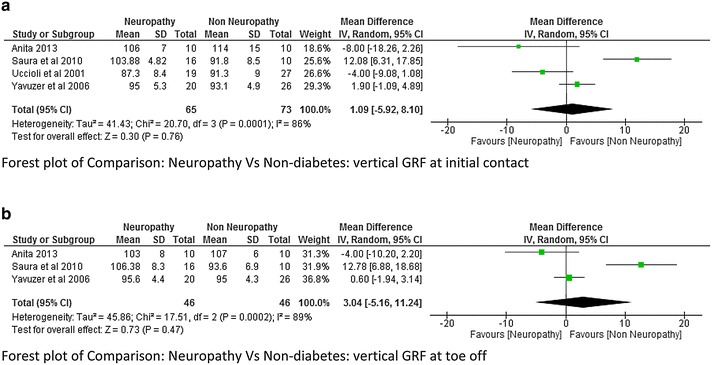
**a** Meta-analysis report for *vertical* ground reaction force at initial contact between neuropathy and non-neuropathy (results do not favor either group). **b** Meta-analysis report for *vertical* ground reaction force at toe off between neuropathy and non-neuropathy (results do not favor either group)

**Fig. 16:**
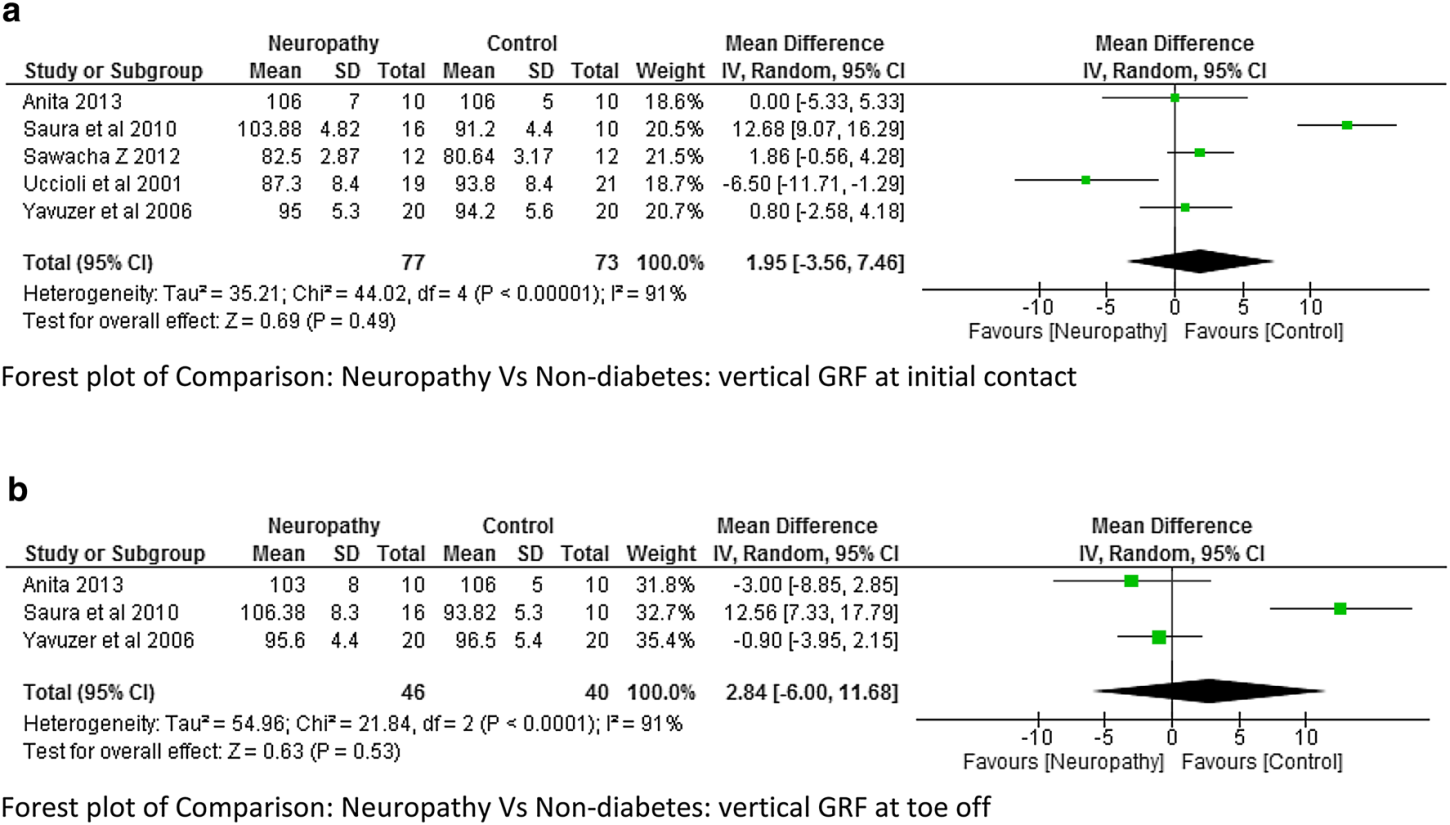
**a** Meta-analysis report for *vertical* ground reaction force at initial contact between neuropathy and non-diabetics (results do not favor either group). **b** Meta-analysis report for *vertical* ground reaction force at initial toe off between neuropathy and non-diabetics (results do not favor either group)

The vertical ground reaction force at initial contact and toe was reported in five studies (Yavuzer et al. 2006; Raspovic 2013; Sawacha et al. 2012; Saura et al. 2010; Uccioli et al. 2001). The Meta analysis report on vertical GRF at initial contact and toe off neuropathy and control group as well as between non-neuropathy and non-diabetes group showed that there was no significant difference. These findings could be seen as there was a lot of variability among the studies while reporting the mean values due to which the heterogeneity was also very high (Figs. 15a, b and 16a, b).

### Joint moment

**Fig. 17:**
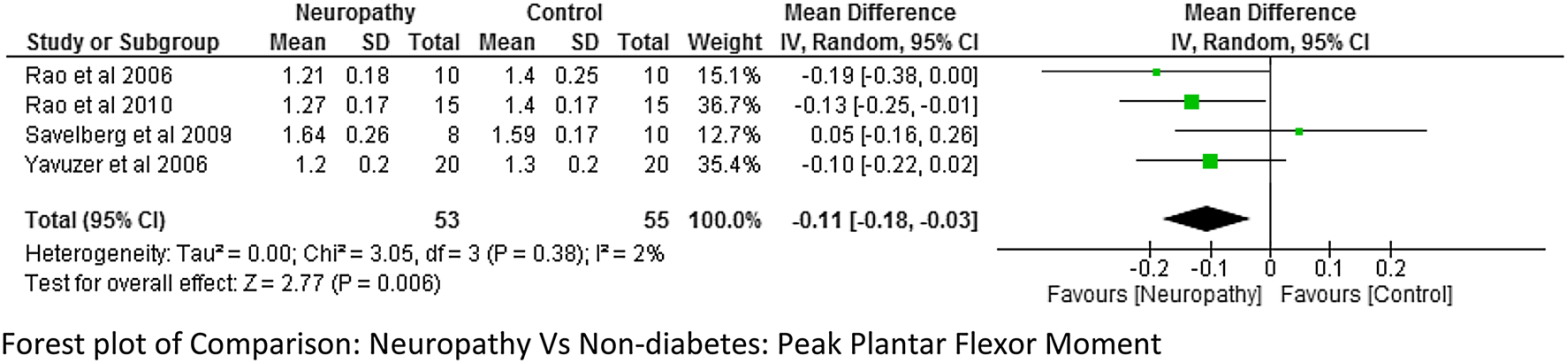
Meta-analysis report for peak plantar flexor moment between neuropathy and non-diabetics (*negative* mean difference represents higher mean values in the second group i.e. non-diabetes)

**Fig. 18:**
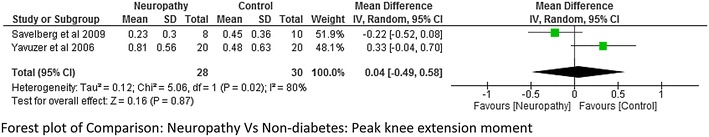
Meta-analysis report for peak knee extension moment between neuropathy and non-diabetics (results do not favor either group)

**Fig. 19:**
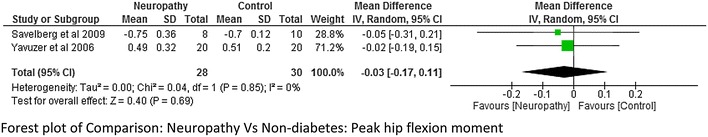
Meta-analysis report for peak hip flexion moment between neuropathy and non-diabetics (results do not favor either group)

Joint flexion/extension moment is one the important kinetic variable for biomechanical analysis. Peak knee and hip joint flexion and extension moment was reported by two studies (Yavuzer et al. 2006; Savelberg et al. 2009). Whereas ankle joint moment was the outcome variable of interest for four studies viz. (Yavuzer et al. 2006; Rao et al. 2010; Savelberg et al. 2009; Rahman et al. 2006). Our meta-analysis report on combining the data from the above studies showed that there was a statistically significant difference between neuropathy and non-diabetes group while reporting peak plantar flexor moment with p = 0.006 and there was minimum heterogeneity among the studies I^2^ = 2 % (Fig. 17). However, only two studies report on hip and knee joint moment it was difficult to establish a significant difference (Figs. 18 and 19).

## Discussion

From the above results and findings it can be said that there were inconsistency and variability among the researchers while reporting the kinetics and kinematics of foot among the comparable groups, though some degree of agreement was seen in reporting certain variables. For easy understanding, it would be relevant to discuss them according to results and findings above. From meta-analysis in Fig. 2 it could be suggested that participants with diabetes and underlying neuropathy walked with slower speed compared to non-diabetes individuals of the same age group. The findings were similar to the previous studies except the study done by Sawacha et al. (2009). The lower walking speed in neuropathy could be seen as a result of motor weakness as well as underlying proprioceptive deficient due to sensory neuropathy (Fernando et al. 2013). Similarly other related Spatio-temporal parameters of gait like stride length was also seen to be lower in neuropathy group. In accordance with findings from previous study, we estimated hip, knee and ankle joint angles to be lower in DPN group when compared to the non-neuropathy and non-diabetes group. The findings from the meta-analysis favored our hypothesis except for hip flexion angle. Two studies study (Yavuzer et al. 2006; Raspovic 2013) reported that maximum hip flexion was reduced in neuropathy group, however contradictory to this one study suggested that hip angle was higher (Gomes et al. 2011). The higher hip flexion angle could be seen as a compensatory mechanism to compensate lower knee and ankle joint range of motion in neuropathy individuals. It could be seen as a gait stabilizing strategy by the neuropathy participants. Looking at kinematics of foot, findings from the studies that focused on the force generation at the hip, knee and ankle and was similar and it was reported that the propelling and braking forces were reduced in the diabetic peripheral neuropathy (DPN) group compared to diabetes mellitus without neuropathy and non-diabetes group (Savelberg et al. 2009). This was expected because we hypothesized that the motor neuropathy leads to proximal and distal muscular weakness of lower extremity (Bansal et al. 2006). However the results regarding the joint moment were inconsistent. The higher values of ankle plantar flexion moment was found in DPN participants by Sawacha et al. (2009), Savelberg et al. (2009) whereas as Yavuzer et al. (2006), Rao et al. (2010) had reported a lower value. The present study and meta-analysis report show that the result was favorable to what reported by Yavuzer et al. (2006) and DPN group had lower mean values. Similarly the results for the knee flexion and extension moments were also inconsistent and a lower degree of agreement was seen among the researchers. The findings could be attributed to different methods and tools used by the researchers. The difference could also be seen as a result of compensatory strategy with knee joint flexion angle. It was reported that the motor component of DPN manifests in a glove and stocking distribution and affects distal joints first (Tesfaye and Selvarajah 2012).

The joint stiffness in diabetic group with neuropathy and non-neuropathic participants was evaluated by Williams et al. (2007). They found that the ankle stiffness in neuropathic group was significantly higher with p value of ≤0.01 at 65–80 % of gait cycle. Unlike ankle, the difference in knee stiffness was found in 50–65 % of gait cycle. The ankle and knee joint stiffness could be a result of motor neuropathy.

The vertical ground reaction force was found to be higher at initial contact in DPN compared to non-neuropathy and non-diabetes participants. At toe off the vertical GRF was found to be high in the study done by Saura et al. (2010) which was just the opposite as reported by Yavuzer et al. (2006). The study done by Sawacha et al. (2012) reported a significant higher value of GRF and Plantar Pressure (PP) at mid-foot and forefoot; this was an important finding as these sites are more prone for ulcers. The present study anticipated that the Vertical GRF in neuropathy would be higher compared non-neuropathy due to neurological and proprioceptive deficit, but unfortunately there was a lot of heterogeneity (I^2^ = 91 %) among the researcher and therefore meta-analysis report was insignificant. This suggests that it would be difficult to say with confidence that neuropathy leads to higher ground reaction force. However individual studies have suggested this fact with greater evidence along with probable reasons. When we look at the plantar pressure distribution, the meta-analysis results suggests that the average plantar pressure, fore-foot pressure, mid-foot pressure were high in neuropathy (Fig. 11 analysis 1.11, Fig. 12 analysis 1.12). Since there are musculoskeletal changes and intrinsic foot muscles become weak, similar results could be expected. It should be noted that high pressure are the most important risk factors for developing foot ulcers, neuropathy individuals are always at a higher risk of developing diabetic foot ulcers at forefoot and mid-foot. The higher plantar pressure in neuropathy could be seen as reduction in plantar tissue thickness in diabetes population. The plantar tissue thickness was reported in two studies (Kumar et al. 2015; Zheng et al. 2006). The former study used the ultrasound indentation system to assess the tissue thickness whereas the other study used the diagnostic ultrasound in a clinical setting. The study reported that there was a significant reduction in the intrinsic foot muscle and tissue thickness in the diabetic group compared to non-diabetic however no significant difference was found between the DPN and non-DPN group.

## Conclusions

The review and the meta- analysis report are of great clinical importance that clearly suggested that there was a significant difference in kinetic and kinematic parameters among the participants with type 2 diabetes mellitus underlying peripheral neuropathy, participants with type 2 diabetes without peripheral neuropathy and non-diabetes participants. Higher values of ground reaction force and plantar pressure has been found in diabetes group with underlying neuropathy which could lead to ulceration and other foot complications. An early screening and analysis of biomechanical alterations in diabetes population can prevent foot complications and subsequent amputation. The review also found that majority of the study had used smaller sample size; therefore a study with larger sample size should be done in order to propose the results more strongly. Based on this review future studies can also be proposed with various interventions to overcome altered foot biomechanics in type 2 diabetes mellitus.
